# The plasticity of gut microbiota contributes to inter-region dietary differences adaptation of ungulates in the Qinghai-Tibet Plateau

**DOI:** 10.1128/msystems.00422-25

**Published:** 2025-07-18

**Authors:** Chengbo Liang, Bin Li, Pengfei Song, Haifeng Gu, Feng Jiang, Meng Zhang, Rui Zhang, Tongzuo Zhang

**Affiliations:** 1 Key Laboratory of Adaptation and Evolution of Plateau Biota, Northwest Institute of Plateau Biology, Chinese Academy of Sciences 34721, Xining, China; 2 College of Life Sciences, University of Chinese Academy of Sciences, Beijing, China; 3 Qinghai Provincial Key Laboratory of Animal Ecological Genomics, Xining, China; 4 College of Eco-Environmental Engineering, Qinghai University, Xining, Qinghai, China; Kobenhavns Universitet, Frederiksberg C, Denmark

**Keywords:** ungulates in the Qinghai-Tibet Plateau, dietary differences, metabolic function, gut microbiota, host adaptation

## Abstract

The gut microbiota in different diets help hosts to obtain sufficient nutrients from food, which is important for ungulates in the Qinghai-Tibet Plateau to adapt to different regions. Quantitatively investigating the relationship between the host diet and the gut microbiota is important for exploring the adaptation of these ungulates to environments. This study used bharals as an example and used high-throughput sequencing techniques and liquid chromatography-mass spectrometry to investigate the diet, the composition, and the function of bharals’ gut microbiotas, as well as the composition of gut microbial metabolites in three regions of the Qinghai-Tibet Plateau. The inter-region differences in them were compared, and relationships among these differences were explored. Results indicated that bharals in the Qinghai Lake basin exhibited a diet predominantly of Polygonaceae and less feeding on Rosaceae and Poaceae compared to bharals in the Kunlun Mountains region and the Sanjiangyuan region. Bharals in the Qinghai Lake basin possessed gut microbiotas with a greater capacity to metabolize carbohydrates. And the functional capacity of such functions was positively correlated with the abundance of Polygonaceae in the diet and negatively correlated with the abundance of Rosaceae and Poaceae. Combined with the lower crude fiber content of Polygonaceae and higher crude fiber content of Rosaceae and Poaceae, we hypothesized that bharals met their energy requirements under low crude fiber intake conditions through their gut microbiota. This study provided insights into understanding how gut microbiotas help hosts adapt to local diets during the adaptation of ungulates to different regions in the Qinghai-Tibet Plateau.

**IMPORTANCE:**

Gut microbiota helping hosts to meet their nutritional requirements in different diets in different regions was thought to underlie the widespread distribution of ungulates in the Qinghai-Tibet Plateau. However, there were few research to prove this. Using bharals as an example, this study quantitatively found that the gut microbiota of ungulates in the Qinghai-Tibet Plateau were more capable of utilizing carbohydrates on a predominantly low-fiber diet. This provides support for the idea that the gut microbiota help hosts adapt to different regions and provides new insights into the role of gut microbes in the adaptation of ungulates to different regions of the Tibetan Plateau.

## INTRODUCTION

Revealing how wildlife adapts to different regions has always been one of the goals of conservation biology. For this, many related elements have received attention. These include how species adapt to food resources in different regions (1). For a wild animal, its adaptation to a region must be based on adaptation to local food resources. Because it needs to obtain the required nutrients from food. As the availability of food affects animals’ diet, there are often inter-region differences in animals’ diet (2, 3). How an animal in different regions can satisfy their nutritional needs in different dietary conditions has become a question worth exploring. As an important participant in food digestion, the gut microbiota can serve as a perspective to explore this issue.

The gut microbiota plays an integral role in food metabolism, nutrient intake, and energy absorption of the host (4, 5). Significant associations between its composition and the host diet had been revealed. For example, the gut microbiota compositions of the muskox (*Ovibos moschatus*) in the Arctic and the chimpanzee (*Pan troglodytes*) in tropical Africa were both significantly correlated with their diet. Muskox with a similar diet had similar gut microbiota, and the microbiota and the diet were negatively correlated in alpha diversity (6). The study on chimpanzees showed that the host diet was more closely linked to the gut microbiota composition than to sex and habitat of hosts (7). These were benefits in understanding the relationship between the gut microbiota and the host diet. Because the gut microbiota composition is partly decoupled from the function (8–10), attention should also be paid to the gut microbiota function when understanding the relationship between the gut microbiota and the host diet. And then further understand how the services that the gut microbiota can provide to the host are linked to the host diet. However, this content remains to be revealed through relevant research.

The Qinghai-Tibet Plateau (QTP) is one of the hotspots for conservation biology research, and there are many rare ungulates here (11). For relevant conservation, how ungulates in the QTP are adapted to different regions had been paid attention to (12, 13). With the requirement of non-invasive sampling, the gut microbiota became an effective way to explore this component. The role of the gut microbiota in the adaptation of ungulates to the QTP had been investigated through local ungulates (14). However, the association between differences in the gut microbiota and differences in the diet among regions of these ungulates remains largely understudied. The present study was conducted to explore this question.

To explore inter-region differences in ungulates’ diets and the accompanying differences in their gut microbiota, it is necessary to study the diet and the gut microbiota of one ungulate species in different regions. The species should be distributed widely in the QTP, and there are differences in the composition of plants and diet of the species across regions. So, bharals (*Pseudois nayaur*) were chosen here. They are distributed widely in and around the QTP (https://www.iucnredlist.org/). The bharal is one of the ungulates with the largest population and the widest distribution range in the QTP (15). Their distribution regions span diverse habitats with different vegetation types (16), involving many ecosystems such as forests, scrublands, grasslands, and deserts (17–20). And it had been shown that there are differences in the diet of bharals in different regions during the same season (21, 22). In the summer, bharals in the Trans-Himalaya region of India fed on 80% and 4% of graminoid herbs and shrubs, respectively (23). These two plant taxa accounted for 51.1% and 20% of the total amount of food taken in Mustang, Nepal, respectively (24). The proportions of the two taxa in the summer bharals’ diet in Manang region, Nepal, were 41.5% and 33.2%, respectively (25). So, choosing bharals as the object can fulfill the requirements of our study. In addition, choosing bharals as the object can also directly provide theoretical support for bharal conservation. Bharal is one of the rare phytophagous ungulates in the QTP and is an important food for rare carnivores such as snow leopards (*Panthera uncia*), leopards (*Panthera pardus*), Eurasian lynx (*Lynx lynx*), and grey wolf (*Canis lupus*) (26–30). Conservation biology issues related to bharals are highly focused on due to the rarity and the important position in the food chain of bharals. Bharals’ environmental adaptations were included. The gut microbiota had been used as a way to study such issues, and differences in the gut microbiota of bharals among seasons and between wild and captive conditions had been identified. These differences included differences in the alpha diversity of the microbiota, differences in the overall composition, differences in the abundance of specific taxa, and differences in the capacity of specific functions. And differences in diet had also been hypothesized to be one influence on these differences (31–34). However, the relationship between differences in the bharals’ diet and differences in the bharals’ gut microbiota is still to be revealed through specialized studies. And the specific situation of this relationship among bharals in different regions in the same season remains largely unknown.

Here, using the bharal as an example, quantitative research methods were employed to analyze the inter-region differences in the diet and the gut microbiota of ungulates in the QTP and explore the relationship between the differences in the diet and the gut microbiota. Bharals’ fecal samples were collected from different regions in the QTP. The bharals’ diet in each region was obtained by determining the composition of *trnL* in fecal samples. Additionally, metagenomic sequencing and untargeted metabolomics were analyzed by fecal samples. Coupled with databases and characterizations of the composition, the function, and the microbial metabolite composition of the bharals’ gut microbiota in each region were obtained. Then the characterizations of the bharals’ gut microbiota in different diets were studied. In order to investigate the potential role of the gut microbiota in utilizing nutrients from different diets in bharals, the metabolic function of the gut microbiota was focused on in our study. Furthermore, the ability of the gut microbiota to produce a certain substance based on metagenomic data and the abundance of that substance based on metabolomic data both reflect the capacity of the gut microbiota to provide that substance to the host. In other words, both the function and the metabolite composition of the gut microbiota reflect how the gut microbiota can serve the host. Based on this, both the function and the metabolite composition were studied here. And the results based on them were expected to be mutually supportive. Based on the above experimental design, the relationships between the bharals’ diet and the composition, the function, and the metabolite composition of the bharals’ gut microbiota were studied. We expect this study to provide a theoretical basis for the conservation of bharals and provide insights for understanding the potential role of the gut microbiota for host adaptation to local diet during the adaptation of ungulates to different regions in the QTP.

## MATERIALS AND METHODS

### Study regions and sampling

**Fig 1:**
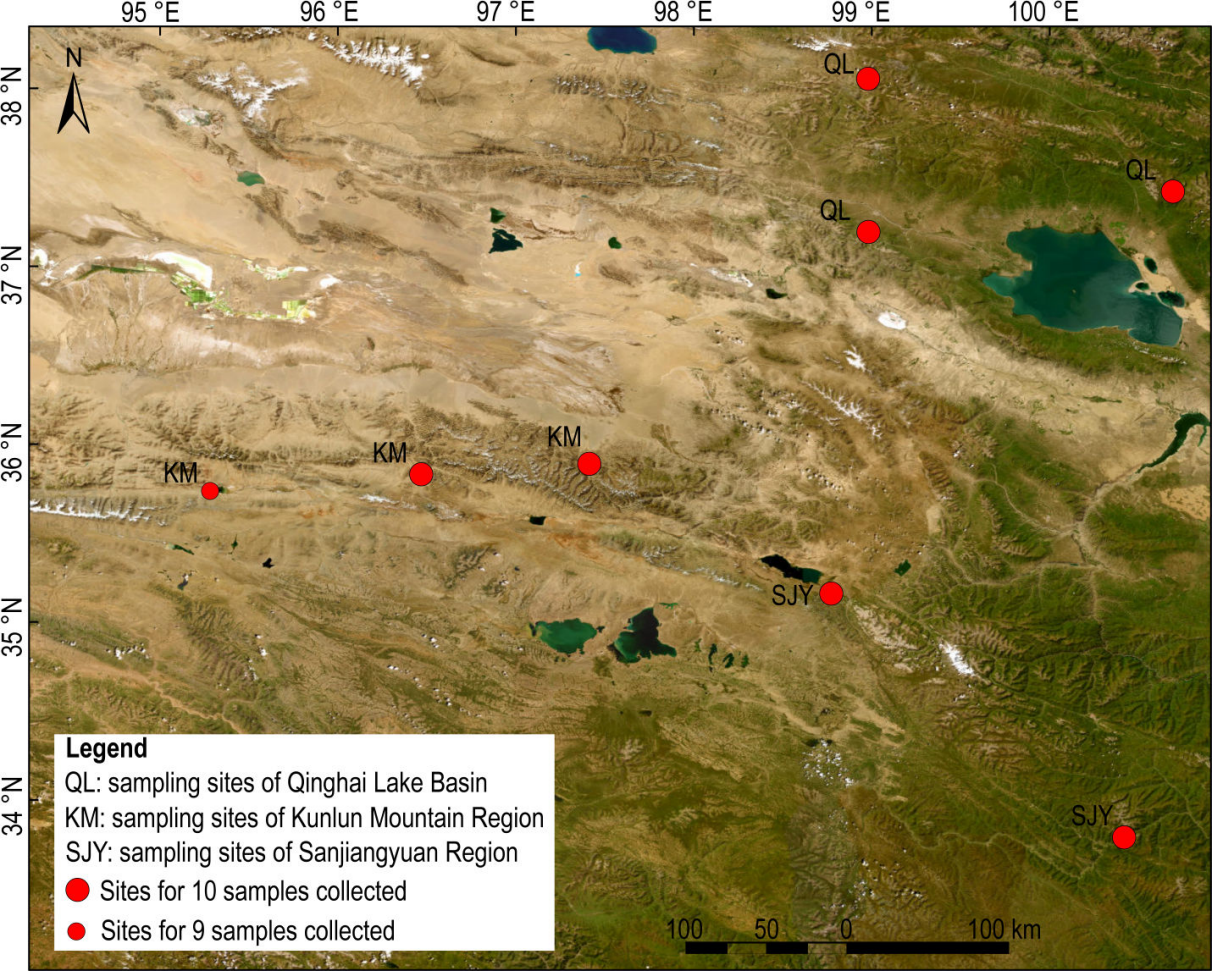
Sampling sites of bharals’ faeces. Service layer credits: source: Esri, Maxar, Earthstar Geographics, and the GIS User Community.

Seventy-nine fresh bharals’ fecal samples were sampled in August 2023. They were sampled in the Qinghai Lake basin (QL) , the Kunlun Mountains region (KM), and the Sanjiangyuan region (SJY), respectively (Fig. 1). The main dominant families of seed plants in all three regions are Poaceae, Asteraceae, and Fabaceae, but there are significant overall differences in the seed plant compositions. Except these three families, the dominant families in the QL are Cyperaceae, Rosaceae, etc. While the KM is dominated by Amaranthaceae, Brassicaceae, etc., the SJY is dominated by Cruciferae, Scrophulariaceae, etc. Furthermore, comparing their plants’ flora revealed that the temperate nature of flora in KM, the SJY, and the QL gradually decreased (17, 35, 36). This inter-region difference in the plant composition meant that the bharals’ diet might differ among the three regions.

### The diet analysis

After extracting DNA from feces by the CTAB method, amplification of the *trnL* gene segment by the universal primers g and h was performed. Subsequently, the libraries were constructed by the TruSeq Nano DNA LT Library Prep Kit, followed by sequencing on the Illumina NovaSeq 6000 platform to obtain raw data.

After quality control and rarefaction by fastp v0.23.4 (37) and QIIME2 (38), the amplicon sequence variant (ASV) table of the diet was obtained. Abundance in the table was expressed as relative abundance. The ASVs were annotated by the National Center for Biotechnology Information nucleotide plant sub-base, the Barcode of Life Data System v4 database, and the self-constructed database of the Innovation Team for Conservation and Management of Animal Diversity in the Tibetan Plateau of the Northwest Plateau Institute of Biology, Chinese Academy of Sciences. A taxonomic category with a lower taxonomic rank and higher identification efficiency was selected to study the bharals’ diet.

Characterizations of the bharals’ diet in regions were analyzed based on the relative abundance of each plant taxa. Inter-region differences in the bharals’ diet were tested using the analysis of similarities (ANOSIM) analysis by the R package vegan v2.6-2 (39). Simpson indexes of regions were calculated by the R package microeco v1.2.2 (40), and differences in this were compared by the Wilcoxon rank sum test. Linear discriminant analysis effect size (LEfSe) analysis was performed using the microeco package in R, selecting taxa with linear discriminant analysis (LDA) scores over 2.5 as the differential plant taxa.

### The composition analysis of the gut microbiota

After extraction of DNA from feces with the FastPure Feces DNA Isolation Kit, the DNA was fragmented by the Covaris M220. After constructing libraries with the NEXTFLEX Rapid DNA-Seq Kit, the samples were subjected to shotgun metagenomic sequencing on the Illumina NovaSeq 6000 platform to obtain raw data. At least 12 GB raw data were obtained from each sample.

The raw data were quality controlled by fastp, and then the reads belonging to the host were fetched by bowtie2 v2.5.4 (41). Then contigs were assembled by megahit v1.2.9 (42, 43), and they were divided into different bins by metabat2 v2.15 (44). The bins with completeness ≥50% and contamination <10% were retained based on the evaluation by checkm v1.2.2 (45). All the retained bins were dereplicated at species level with 95% average nucleotide identity by drep v3.5.0 (46), respectively. The taxonomy label of species-level genome bins (SGBs) was predicted by gtdbtk v2.4.0 (47), and the abundance of SGBs in each sample was obtained by metawrap v1.3.2 (48).

Inter-region differences in the composition of the bharals’ gut microbiota were tested by the ANOSIM analysis. Simpson indexes and connectance of microbial co-occurrence network were calculated by the R package microeco and igraph v1.5.1 (49) respectively. Inter-region differences in these were compared by the Wilcoxon rank sum test. Taxa with LDA scores over 2.5 were selected as differential microbial taxa by the LEfSe analysis.

### The function analysis of the gut microbiota

The raw data were quality controlled by fastp, and then the reads belonging to the host were fetched by bwa v0.7.17 (50). Then contigs were assembled by megahit. After predicting open reading frames from contigs by Prodigal v2.6.3 (51), a non-redundant gene set was generated by cd-hit v4.6.1 (52). Finally, abundance information of each gene in each sample was obtained by SOAPaligner v2.21 (53), with abundance measured in transcripts per million. The obtained genes were compared with functional databases using DIAMOND v2.0.13 (54) and HMMER v3.1b2 (55). The Evolutionary genealogy of genes: Non-supervised Orthologous Groups (eggNOG) database, the Kyoto Encyclopedia of Genes and Genomes (KEGG) database, and the Carbohydrate-Active enZYmes (CAZy) database were used. The annotation results and abundance information were combined to obtain the function of the bharals’ gut microbiota. To explore the inter-region differences in the metabolic function of the gut microbiota, metabolic functions in the Clusters of Orthologous Genes (COG) database, metabolic pathways of level three and enzymes in the KEGG database, and families in the CAZy database were selected for the following analyses.

Inter-region differences in the metabolic function of the bharals’ gut microbiota were tested by the ANOSIM analysis. Functions in the COG database, pathways and enzymes in the KEGG database, and families in the CAZy database with LDA scores over 2.5 were selected as differential functions by the LEfSe analysis. For the results based on the CAZy database, the glycoside hydrolase (GH) enzyme families that show significant inter-region differences were focused on. The abundance of differential families across regions was compared by the Wilcoxon rank sum test. GHs that showed significantly higher abundance in each region compared to the others were selected based on these results. The enzymes in these families were obtained from the CAZy database. The enzymes common to the groups were then removed, and it was determined which reactions were catalyzed by enzymes specific to each group based on the ENZYME (Enzyme nomenclature) database. In addition, the enrichment analysis based on the KEGG database was performed. Pathways of level 3, where the absolute value of the reporter score was over 1.65, were focused on.

### Analysis of gut microbial metabolite

Approximately 50 mg of each fecal sample was taken and analyzed by liquid chromatography-mass spectrometry using a UHPLC-Q Exactive HF-X system to obtain raw data. Raw data were processed with Progenesis QI to obtain the total normalized abundance of each metabolite in each sample, and metabolites were annotated by the KEGG database and the Human Metabolome Database. Then the microbial metabolites were screened with MetOrigin (56) for the following analysis.

Inter-region differences in the metabolite composition of bharals’ gut microbiota were tested by the ANOSIM analysis. The orthogonal partial least squares discriminant analysis (OPLS-DA) analysis was run by the R package ropls v1.6.2 (57) to select metabolites with VIP_pred_OPLS-DA over 1 as the differential metabolites and to compare the inter-region abundance of these metabolites.

### Data correlation analysis

To explore the relationships among the diet, the microbiota composition, the metabolic function of the microbiota, and the microbial metabolites composition, their correlation with each other was calculated. Inter-sample differences in them were calculated by vegan packet. The relationship of differences in the microbiota composition to dietary differences, the relationship of dietary differences and differences in the microbiota composition to differences in the metabolic function of the microbiota, and the relationship of dietary differences and differences in the microbiota composition to differences in the composition of microbial metabolites were analyzed using the multiple regression on distance matrices (MRM) models by the R package ecodist v2.1.3 (58).

In addition, the relationships among differential plant taxa, differential microbial taxa, differential metabolic functions, and differential metabolites were focused on. They were *z*-score normalized by the scale function in R. The relationships between differential plant taxa and differential microbial taxa, the relationships between differential plant taxa and differential metabolic functions, the relationships between differential microbial taxa and differential metabolic functions, the relationships between differential plant taxa and differential metabolites, and the relationships between differential microbial taxa and differential metabolites were explored by the Spearman correlation analysis.

## RESULTS

### Dietary differences

Eventually, 247 high-quality ASVs were retained, which belonged to one phylum, four classes, 22 orders, 40 families, 94 genera, and 108 species. Except at the phylum level, the identification efficiencies at other taxonomic categories were not 100%, with the highest efficiency of 87.5% at the family level (Table S1). The following studies of diet were conducted at the family level because its taxonomic ordinal rank was low and the identification efficiency at this level was high.

**Fig 2:**
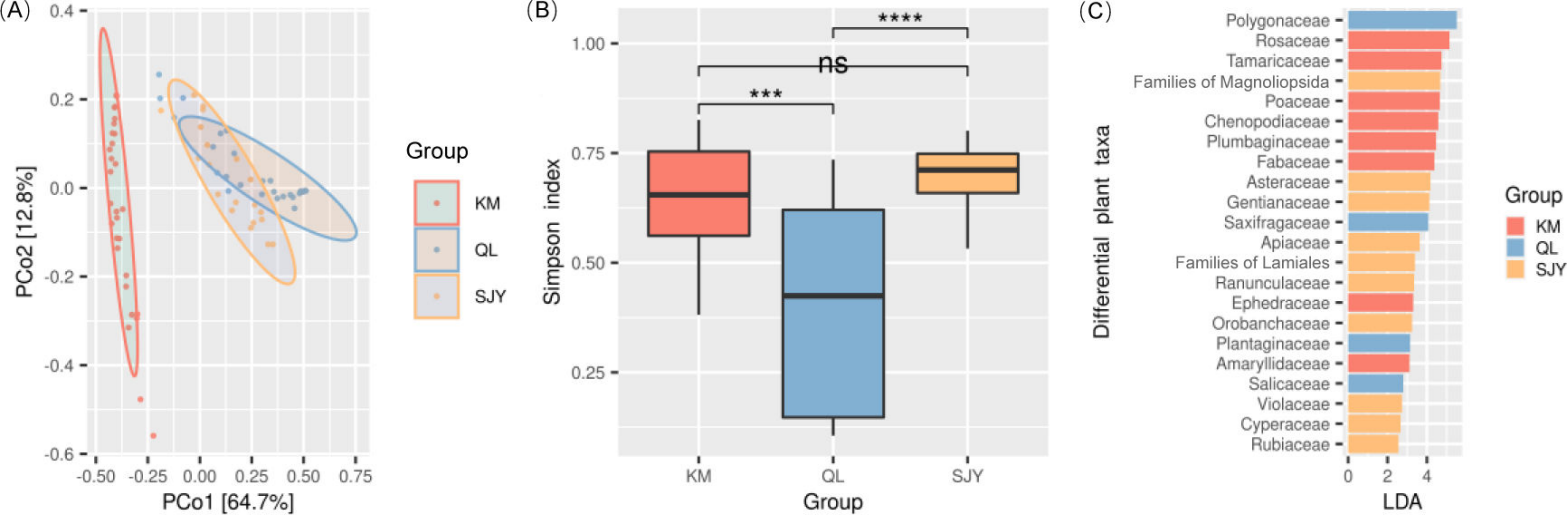
Dietary differences among regions. (**A**) Differences in the beta diversity of the bharals’ diet among regions. (**B**) Differences in the alpha diversity of the bharals’ diet among regions. “ns”: *P* > 0.05, “***”: *P* < 0.001, “****”: *P* < 0.0001. (**C**) Differential plant taxa in the bharals’ diet among regions.

There were significant inter-region differences in the bharals’ diet (Fig. 2A; Table S2). The alpha diversity of the bharals’ diet in the QL was lower than that of other regions (Fig. 2B). Local bharals mainly fed on Polygonaceae, whose relative abundance is 66.85%. Bharals in the KM mainly fed on Rosaceae and Poaceae, with a cumulative relative abundance of 60.46%. Bharals in the SJY mainly fed on Polygonaceae and Rosaceae, with a cumulative relative abundance of 63.71% (Table 1). Totally, 22 families with inter-region differences were identified as differential plant taxa by the LEfSe analysis. The LDA scores of Polygonaceae, Rosaceae, and Poaceae, the three plant taxa that bharals mainly fed on, were ranked first, second, and fifth high, respectively (Table S3; Fig. 2C). Their inter-region differences effectively explained inter-region differences in the bharals’ diet.

**TABLE 1:** Most prevalent dietary plant taxa at the family level^*a*^

Plant taxa	Rank in QL	Rank in KM	Rank in SJY	Average relative abundance in QL	Average relative abundance in KM	Average relative abundance in SJY
Polygonaceae	1	15	1	66.85%	0.11%	43.68%
Rosaceae	2	1	2	19.03%	47.23%	20.03%
Poaceae	3	2	4	4.44%	13.23%	8.40%
Saxifragaceae	4	22	18	2.33%	0.01%	0.08%
Fabaceae	5	5	5	1.72%	6.44%	5.42%
Crassulaceae	6	11	8	1.68%	0.43%	1.61%
Asteraceae	7	7	6	1.61%	2.97%	4.71%
Tamaricaceae	28	3	35	0.01%	11.18%	0.00%
Chenopodiaceae	26	4	32	0.01%	7.47%	0.02%
Plumbaginaceae	19	6	27	0.04%	6.06%	0.04%
Families of Magnoliopsida	12	8	3	0.26%	1.71%	9.72%
Primulaceae	22	9	13	0.02%	1.11%	0.22%
Gentianaceae	23	27	7	0.02%	0.00%	2.57%

^*a*^ Note: families with an average relative abundance of over 1% in each region were listed. QL: Qinghai Lake basin. KM: Kunlun Mountains region. SJY: Sanjiangyuan region.

### Differences in the gut microbiota composition and the relationship between it and the diet

**Fig 3:**
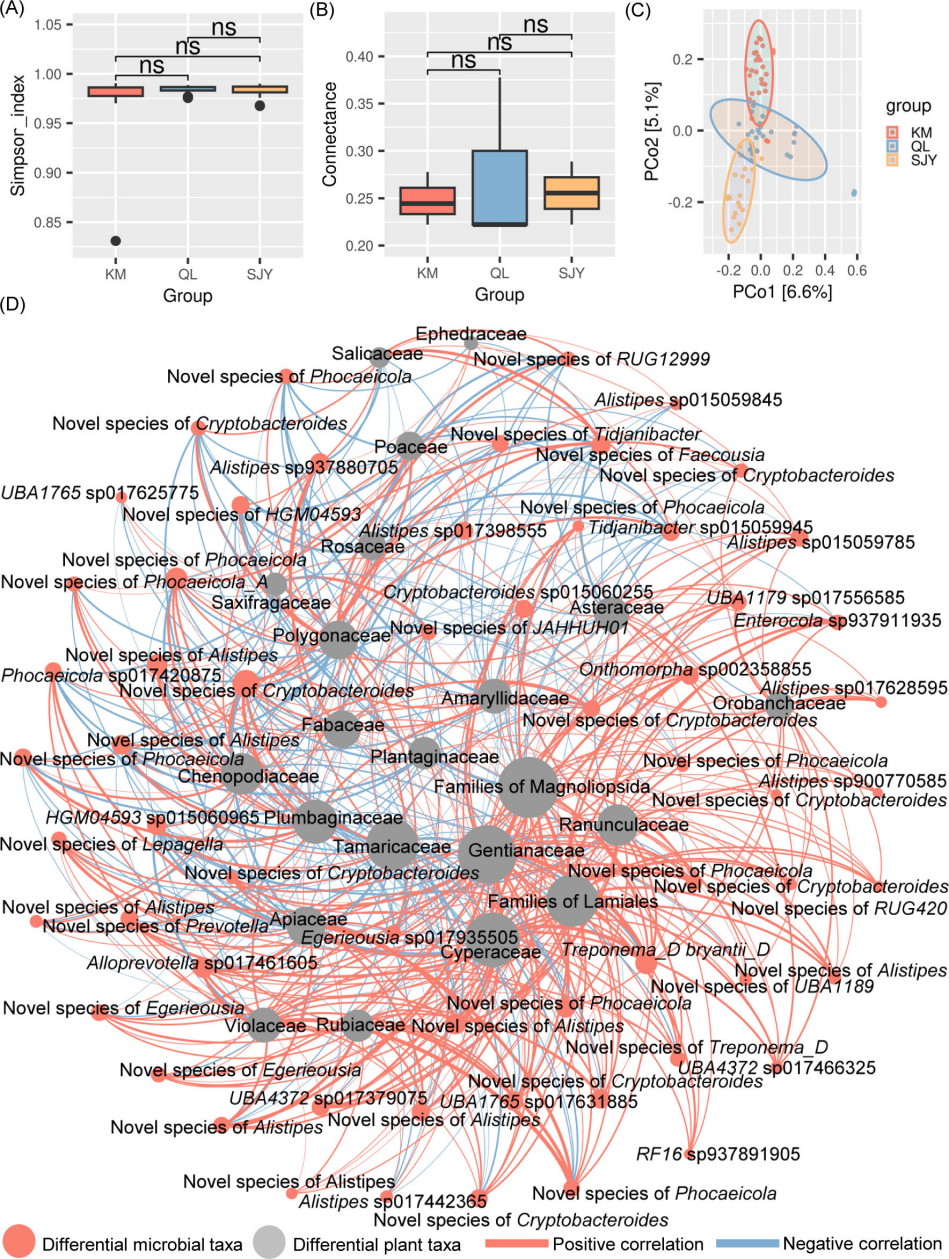
Differences in characterizations of the bharals’ gut microbiota among regions and correlation between differential microbial taxa and differential plant taxa. (**A**) Differences in the alpha diversity of the bharals’ gut microbiota among regions. “ns”: *P* > 0.05. (**B**) Differences in the connectance of the bharals’ gut microbiota co-occurrence network among regions. “ns”: *P* > 0.05. (**C**) Differences in the beta diversity of the bharals’ gut microbiota among regions. (**D**) Correlation between differential microbial taxa and differential plant taxa. Larger nodes indicate more taxa associated with them. A thicker line indicates a stronger correlation between the two nodes of the line.

The annotation of SGBs obtained 3,134 species-level taxa. The bharals’ gut microbiota did not differ significantly among regions in terms of alpha diversity and co-occurrence network complexity (Fig. 3A and B). However, the main species in each region were different (Table 2), although these microorganisms all belong to the main gut microbial taxa of ungulates in the QTP, Bacillota, Fibrobacterota, Spirochaetota, or Bacteroidota (59–62). And there were significant inter-region differences in the overall microbiota composition (Fig. 3C; Table S2). Totally, 60 species-level taxa with inter-region differences were identified as differential microbial taxa by the LEfSe analysis (Table S3).

**TABLE 2:** Most prevalent gut microbial taxa at the species level^*a*^

Microbial taxa	Rank in QL	Rank in KM	Rank in SJY	Average relative abundance in QL	Average relative abundance in KM	Average relative abundance in SJY
*Cryptobacteroides* sp017556765	1	10	2	1.66%	0.71%	1.43%
Novel species of *UBA1189*	2	103	83	1.29%	0.23%	0.28%
*Alistipes* sp017416915	3	71	21	1.11%	0.29%	0.53%
Novel species of Lachnospiraceae	4	1,550	2,215	0.95%	0.00%	0.00%
*Cryptobacteroides* sp017461285	5	28	28	0.84%	0.44%	0.48%
Novel species of *Cryptobacteroides*	8	3	191	0.75%	1.03%	0.15%
*Alistipes* sp937880705	739	1	79	0.02%	1.13%	0.29%
Novel species of *Alistipes*	274	2	172	0.10%	1.12%	0.16%
Novel species of *Cryptobacteroides*	2,369	4	56	0.00%	1.00%	0.34%
*HGM04593* sp015060965	33	5	411	0.46%	0.95%	0.06%
*Alistipes* sp015060115	19	8	1	0.55%	0.79%	1.82%
*UBA1765* sp017631885	3,097	3,100	3	0.00%	0.00%	1.37%
*UBA4372* sp017628845	3,132	3,132	4	0.00%	0.00%	1.11%
*Treponema_D bryantii_D*	181	557	5	0.14%	0.04%	1.09%

^*a*^ Note: the top 5 species in terms of relative abundance in each region were listed. QL: Qinghai Lake basin. KM: Kunlun Mountains region. SJY: Sanjiangyuan region.

According to the results of the MRM model, differences in the microbiota composition were significantly correlated with the diet (Table 3). The abundance of specific differential microbial taxa correlates with the abundance of specific differential plant taxa (Fig. 3D). Among the 22 differential plant taxa, Polygonaceae, Rosaceae, and Poaceae, the main food of bharals, were significantly associated with 31 differential microbial taxa. They were significantly associated with 27, 19, and 19 differential microbial taxa, respectively.

**TABLE 3:** Correlates of the gut microbiota composition, the metabolic functions, and the metabolite composition

Formula	Explanatory variable	Regression coefficient	*P*	*R* ^2^
Composition of microbiota ~ diet	Diet	0.3071	0.0001	0.0943
Functions based on eggNOG database ~ composition	Composition	0.5182	0.0001	0.2686
Functions based on pathways of KEGG database ~ diet + composition	Diet	0.0897	0.0001	0.1442
	Composition	0.3424	0.0372	
Functions based on enzymes of KEGG database ~ composition	Composition	0.3747	0.0001	0.1404
Functions based on CAZy database ~ composition	Composition	0.5399	0.0001	0.2915
Metabolite ~ diet + composition	Diet	0.4092	0.0001	0.4747
	Composition	0.4426	0.0001	

### Differences in the metabolic function of the microbiota and the relationship of the diet and the microbiota composition to it

**Fig 4:**
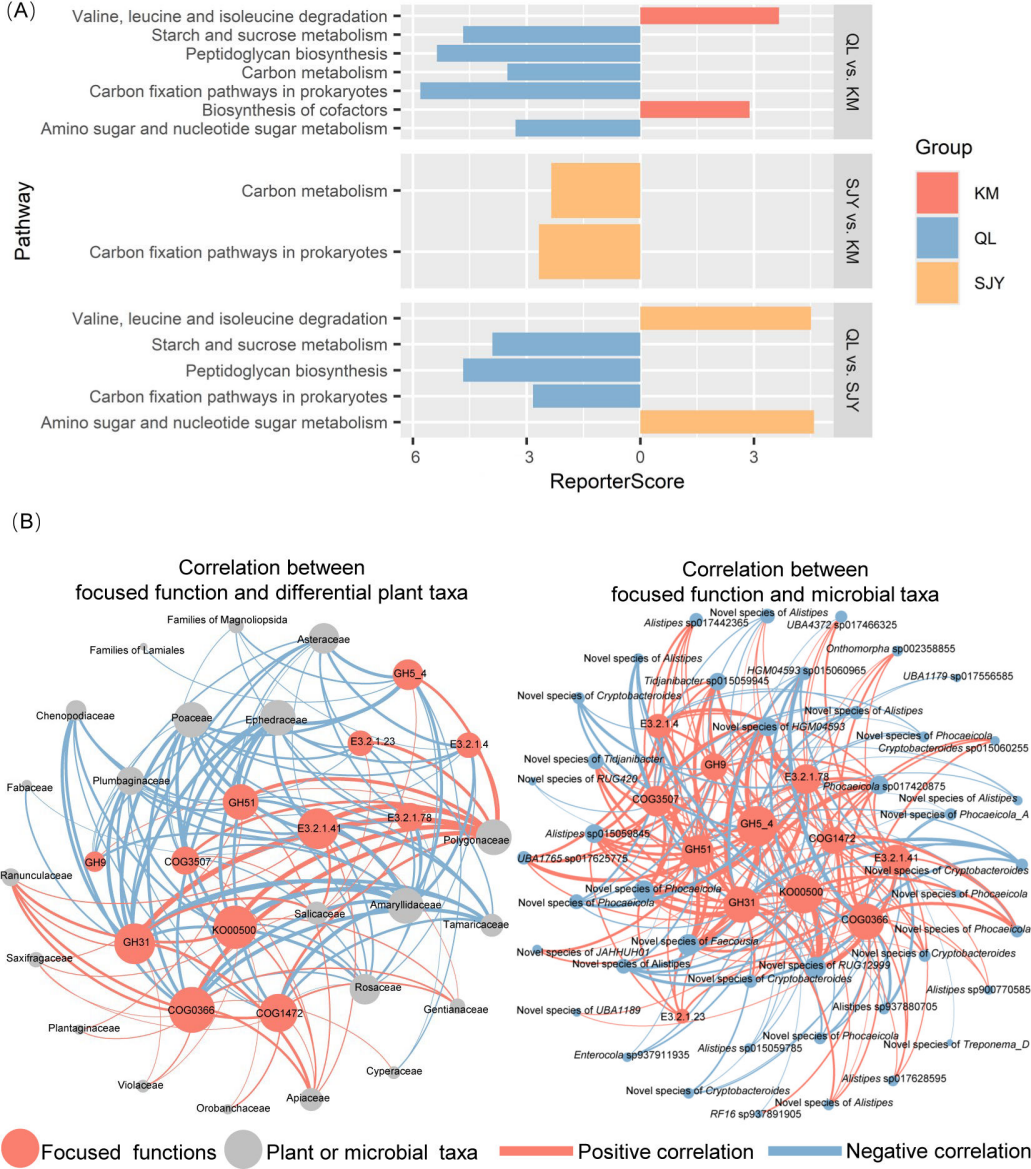
Differential function and their correlation between differential plant taxa and differential microbial taxa. (**A**) Differential function based on the KEGG enrichment analysis (*P* < 0.05). QL: Qinghai Lake basin. KM: Kunlun Mountains region. SJY: Sanjiangyuan region. (**B**) Correlation between differential plant taxa and differential microbial taxa to the focused functions. Larger nodes indicate more functions or taxa associated with them. A thicker line indicates a stronger correlation between the two nodes of the line. KO00500: starch and sucrose metabolism; COG3507: beta-xylosidase; COG1472: periplasmic beta-glucosidase and related glycosidases; COG0366: glycosidase/amylase.

According to results based on the COG databases, KEGG databases, and CAZy databases, there were significant inter-region differences in the overall metabolic function of the microbiota (Table S2). There were significant inter-region differences in specific metabolic functions. Fourteen differential metabolic functions were identified by the LEfSe analysis based on the COG database. Among them, the “beta-xylosidase,” “glycosidase/amylase,” and “periplasmic beta-glucosidase and related glycosidases” had higher abundance in the QL (Table S3). Beta-xylosidase and beta-glucosidase are involved in the utilization of xylan (an important component of hemicellulose) and cellulose, respectively. These results implied that the bharals’ gut microbiota in the QL had a higher capacity to use cellulose, hemicellulose, xylan, and starch. Nine differential metabolic pathways were identified by the LEfSe analysis based on the KEGG database. Among them, the abundance of “starch and sucrose metabolism” was higher in the QL than in other regions (Table S3). This corresponded to the results based on the COG database and was also supported by the results of the enrichment analysis (Fig. 4A). According to the result of the enrichment analysis, the higher abundance of “starch and sucrose metabolism” in the QL was attributed to the fact that some genes, such as *amyA*, *bglB*, *nplT*, *cgt*, *glgA*, *glk*, *malL*, *pgi1*, *SGA1*, and *susB*, had a higher abundance in QL than their abundance in the other two groups. In addition, eight differential metabolic enzymes were identified by the LEfSe analysis based on the KEGG database (Table S3). Five of them are glycosidases. Among these five glycosidases, four glycosidases had higher abundance in the QL. The four glycosidases, E3.2.1.4, E3.2.1.23, E3.2.1.41, and E3.2.1.78, can catalyze the hydrolysis of mannans, galactomannans, glucomannans, amylum, cellulose, and some compounds that can be hydrolyzed to produce galactose. This result also implied that the bharals’ gut microbiota in the QL had a higher capacity to use saccharides.

Thirty-eight carbohydrase families were identified as differential families by LEfSE analysis based on the CAZy database. Among them, nine, five, and one GHs had significantly higher abundance in the QL, KM, and SJY, respectively (Fig. S1). Nine differential GHs with high abundance in the QL include a variety of unique enzymes. They can catalyze the conversion of arabinoxylan, arabino-oligosaccharides, lichenan, galactomannan, xylan, hardwood xylan, glucomannan, chitin, cellulose, and other complex compounds into short-chain polysaccharides and oligosaccharides or catalyze the conversion of reaction products into glucose, xylose, galactose, mannose, arabinose, fructose, and sugar fermentation intermediates. These enzymes mainly belonged to four GHs, GH31, GH51, GH5_4, and GH19. There were fewer unique enzymes in the five differential GHs that had higher abundance in the KM. The unique enzymes can catalyze the hydrolysis of specific sugar-containing base compounds. There was only one unique enzyme in the one differential GH that had higher abundance in the SJY. It can catalyze oligosaccharides into fucose (Table S4). These results implied that the bharals’ gut microbiota in the QL had a higher energy utilization efficiency of polysaccharides and oligosaccharides compared to the microbiota in the other two regions.

According to the results of MRM analyses based on the COG databases, KEGG databases, and CAZy databases, the metabolic function of the bharals’ gut microbiota was always significantly correlated with the composition of the bharals’ gut microbiota. The bharals’ diet correlated with the metabolic function of the bharals’ gut microbiota only when based on the pathways of the KEGG database. And the correlation between the composition and the metabolic function of the microbiota was higher compared to the correlation between the diet and the metabolic function of the microbiota (Table 3). Based on the analysis of inter-region differences in the metabolic function, the relationships of differential microbial taxa and differential plant taxa to 12 functions, “beta-xylosidase,” “glycosidase/amylase,” “periplasmic beta-glucosidase and related glycosidases,” “starch and sucrose metabolism,” GH31, GH51, GH5_4, GH19, E3.2.1.4, E3.2.1.23, E3.2.1.41, and E3.2.1.78 were focused on. Among the 22 differential plant taxa, 21 taxa were each significantly associated with varying numbers of the focused functions (Fig. 4B). Among the three plant families that were the main food for bharals, there is a significant positive correlation among the abundance of Polygonaceae and the abundance of all focused functions. The abundance of Rosaceae and Poaceae was significantly correlated with the abundance of some focused functions, and the correlations among them were all negative. Among the 60 differential microbial taxa, 42 taxa were each significantly associated with varying numbers of the focused functions (Fig. 4B). Among the 42 taxa, 14 taxa, *UBA1765* sp017625775, one novel species of *JAHHUH01*, *Alistipes* sp017442365, *Cryptobacteroides* sp015060255, one novel species of *Tidjanibacter*, one novel species of *Cryptobacteroides*, *Phocaeicola* sp017420875, *Tidjanibacter* sp015059945, one novel species of *Phocaeicola*, one novel species of *HGM04593*, *Alistipes* sp015059845, one novel species of *RUG12999*, one novel species of *Faecousia*, and one novel species of *Cryptobacteroides* had higher abundance in the QL. The significant correlations between the 14 taxa and the focused function were all positive. The other 28 taxa had higher abundance in the KM or SJY. The significant correlations between the 28 taxa and the focused function were almost negative.

### Differences in the composition of microbial metabolites and the relationship of the diet and the microbiota composition to it

**Fig 5:**
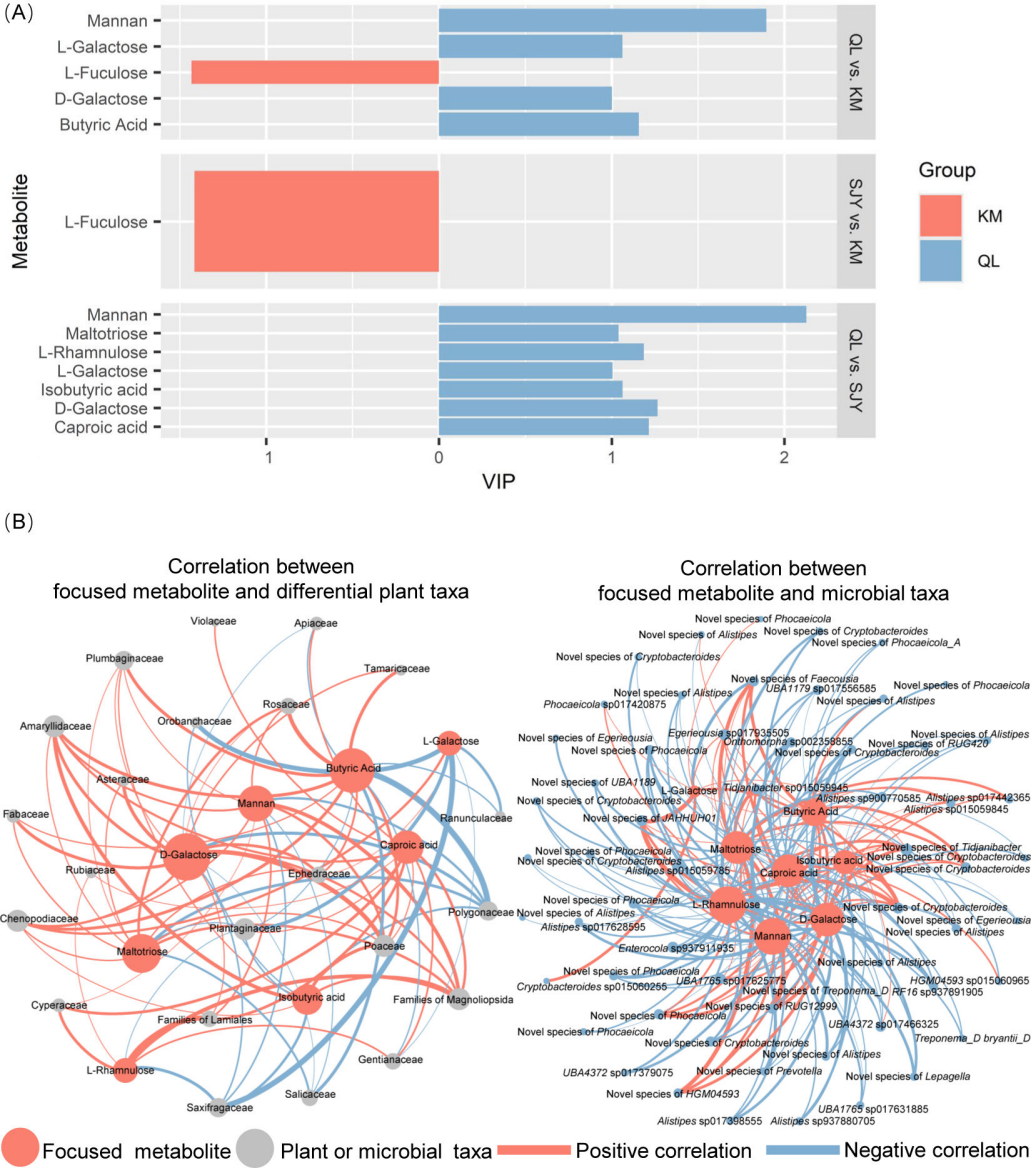
Focused metabolites and their correlation between differential plant taxa and differential microbial taxa. (**A**) Focused function based on OPLS-DA analysis (*P* < 0.05). QL: Qinghai Lake basin. KM: Kunlun Mountains region. SJY: Sanjiangyuan region. (**B**) Correlation between differential plant taxa and differential microbial taxa to the focused metabolites. Larger nodes indicate more metabolites or taxa associated with them. A thicker line indicates a stronger correlation between the two nodes of the line.

A total of 733 microbial metabolites were detected. There were significant inter-region differences in the overall composition of microbial metabolites (Table S2). OPLS-DA analysis showed that 357 metabolites differed among regions, including nine sugars and short-chain fatty acids (Fig. 5A). Among the nine metabolites, the abundances of mannan, L-galactose, and D-galactose in the QL were higher than their abundance in the other two regions. The abundance of butyric acid in the QL was higher than that in the KM. The abundance of L-rhamnulose, maltotriose, caproic acid, and isobutyric acid in the QL was higher than that in the SJY. The high abundance of these oligosaccharides, monosaccharides, and short-chain fatty acids in the QL indicated that the bharals’ gut microbiota in the QL had a greater capacity to utilize the energy from polysaccharides than the microbiota in other regions. To some extent, these results were supported by results of inter-region differences in the metabolic function of the microbiota. The inter-region differences in metabolites also indicated that the abundance of L-fuculose was higher in the KM than in other regions. However, the results did not support each other with the results of the differences in the metabolic function.

MRM analysis showed that the composition of microbial metabolites was correlated with the microbiota composition and the diet. The correlation between the composition of the microbiota and metabolites was similar to that between the diet and the metabolite composition (Table 3). Among the nine sugars and short-chain fatty acids with inter-region differences, the differential patterns of eight metabolites, excluding L-fuculose, were consistent with differences in the metabolic function. Therefore, the correlations of these eight metabolites with specific microbial and plant taxa were focused on. Among the 22 differential plant taxa and 60 differential microbial taxa, significant correlations were observed with varying numbers of the focused metabolites (Fig. 5B). The number of metabolites significantly associated with the five plant taxa, Poaceae, Amaryllidaceae, Chenopodiaceae, families of Magnoliopsida, and Plantaginaceae, was the highest, with each having six metabolites showing significant correlation. In the three plant families mainly fed on by bharals, the abundance of Polygonaceae was significantly correlated with the abundance of some focused metabolites, and the correlations among them were all positive. The abundance of Rosaceae and Poaceae was significantly correlated with the abundance of some focused metabolites, and the correlations among them were all negative. The 14 microbial taxa, which had higher abundance in the QL and were positive with the focused function, were all positive with the focused metabolites.

## DISCUSSION

### The bharals’ diet

The results of the bharals’ diet indicated that bharals mainly fed on Polygonaceae in the QL, mainly fed on Rosaceae and Poaceae in the KM, and mainly fed on Polygonaceae and Rosaceae in the SJY. The summer bharals’ diets in the KM and the Chang Tang region were similar, with Rosaceae dominating in both regions (21). Both areas exhibit a temperate nature in terms of the composition of seed plants. The dominant top 7 families in both areas include Poaceae, Asteraceae, Fabaceae, Amaranthaceae, Brassicaceae, Ranunculaceae, and Rosaceae (63–66). Similarities in the environment and the floristic composition may provide a basis for similarities in the summer bharals’ diet in the two regions. The bharals’ diets in the QL and the SJY differ from all the previous studies in other regions, which may be attributed to the unique local environments in the two regions (21–25). As a species with a broad dietary range, bharals in different environments can meet their nutritional needs by consuming different plants (22). In the two regions with unique environments, the bharals had the unique diet.

In all three study regions, bharals’ main food was not the dominant local plant families of Poaceae, Asteraceae, and Fabaceae. This may be related to bharals’ habitat preferences and interspecific competition with other ungulates. To avoid predators and harsh weather, bharals prefer cliffs and steep slopes (67), especially the upper and middle parts of these habitats near the bare rock (18, 68). Bharals are good at jumping and climbing. In these habitats, bharals can flee quickly when encountering predators. The color near the bare rock is similar to the color of the bharals’ fur, and the bharals’ activities here can serve as concealment from their predators (69). In addition, the bare rock acts as a windbreak, which facilitates bharals' insulation (18). Although they may be active in other habitats in order to consume enough food and water, bharals are mainly active around these habitats (69, 70). In this context, plants in these habitats and nearby habitats are more accessible to bharals than plants in other habitats in the distribution range. This may result in the bharals’ diet being influenced more by the floristic composition of the habitats described above and less by the floristic composition of the whole region. Then bharals will be less likely to feed on some plants that are predominantly distributed in other habitats, even if they are the main dominant plants for the whole region. And the dominant plants in the above habitats may be taken more frequently by bharals, even if they are not clearly dominant for the whole area. In addition to bharals’ own preferences, the activities of sympatric ungulates can also affect bharals’ activities, which can affect the bharals’ diet (18, 69, 71–73). This may also be the reason why bharals do not mainly feed on dominant plants in study regions. Bharals are sympatric with many domestic or wild ungulates, such as sheep (*Ovis aries*), yak (*Bos grunniens*), and argalis (*Ovis ammon*), in the study area, and they compete with each other to some extent (74, 75). In order to mitigate the competition, these ungulates exhibit a degree of temporal and spatial separation in their use of habitat. For example, they use different habitats or use the same habitat at different times (18, 23, 25). Habitat available to bharals and the amount of time they are available for specific habitats may be affected by the influence of other ungulates. The plant resources they can meet and the time they can take specific plants are consequently affected. Then the bharals’ diet may be affected. The above influences may collectively cause bharals not to use the main dominant plants in their distribution range as their main food.

### Differences in the bharals’ gut microbiota in different diets

In different diets, differences in the bharals’ gut microbiota primarily manifested in the abundance differences of specific taxa, functions, and metabolites, whereas there was a lack of notable differences in the overall characteristics of the microbiota. Among the bharals’ gut microbiota in three regions, there were no significant differences observed in alpha diversity and co-occurrence network complexity. But the dominant species within the microbiota, the overall situation of the bharals’ gut microbiota in the composition, the metabolic function, and the metabolite composition varied among regions, and abundance of some microbial taxa, metabolic functions, and metabolites differed significantly among regions. Results of MRM models revealed the relationship among the overall situation of the four things: the diet, the microbiota composition, the metabolic function, and the metabolite composition. The results showed that the microbiota composition, the metabolic functions, and the metabolite composition varied with dietary changes. Among them, both the metabolic function and the metabolite composition were related to the diet and the microbiota composition. The microbiota composition was more closely linked to the metabolic function than the diet. And both were similarly close to the metabolite composition. This reflected the relationship between the host diet, the gut microbiota composition, the metabolic function of the gut microbiota, and the metabolite composition of the gut microbiota. The metabolic function of the gut microbiota is the collection of metabolic functions of various microbial taxa in the gut microbiota. The microbiota composition is decisive for the metabolic function of the microbiota, and the diet only influences the metabolic function to a certain extent by influencing the microbiota composition (9, 76). Thus, the microbiota composition was more closely related to the metabolic function of microbiota than to the diet in bharals. While the metabolites of the gut microbiota are the products of the nutrients in the food ingested by the host after the gut microbiota has performed its metabolic function. Both the host diet and the metabolic function of the gut microbiota play a role in determining its composition. So, they were similarly close to the metabolite composition of the gut microbiota.

The inter-region differences in each metabolic function and metabolite of the gut microbiota showed that, in the QL, the bharals’ gut microbiota had a greater capacity to utilize carbohydrates than the microbiota in the other two regions. The functional differences based on the metagenomic data indicated that the gut microbiota of bharals in the QL had a greater capacity to convert macromolecular carbohydrates into small-molecule sugars that are more readily utilized by bharals than the microbiota in the other two regions. Differences in metabolite composition based on the metabolomic data indicated that the metabolites of the bharals’ gut microbiota were higher in small-molecule sugars and short-chain fatty acids in the QL compared to those in the other two regions. All these substances are metabolites of macromolecular carbohydrates. They are more easily utilized by bharals than macromolecular carbohydrates. These differences in the function and the metabolite composition of the microbiota suggested that the gut microbiota of bharals in the QL might provide greater assistance to bharals in utilizing dietary carbohydrates than in the other two regions. These corresponded to the bharals’ dietary characteristics in the three regions. Compared with the other two regions, the most important feature of the diet of bharals in the QL was that there were more Polygonaceae and less Rosaceae and Poaceae. Polygonaceae tends to have lower crude fiber content than Rosaceae and Poaceae (77, 78). When taking food with fewer nutrients, bharals need to digest the food more thoroughly to meet their energy needs (79). We hypothesize that the greater carbohydrate utilization capacity of the gut microbiota of bharals in the QL provides assistance in adapting bharals to the local diet with low crude fiber content. Combining the differences in diet, gut microbiota composition, and gut microbiota function among the three regions, the dietary intake of bharals in the QL was lower in crude fiber. In this diet scenario, the bharals’ gut microbiota had higher abundance of *UBA1765* sp017625775, one novel species of *JAHHUH01*, *Alistipes* sp017442365, *Cryptobacteroides* sp015060255, one novel species of *Tidjanibacter*, one novel species of *Cryptobacteroides*, *Phocaeicola* sp017420875, *Tidjanibacter* sp015059945, one novel species of *Phocaeicola*, one novel species of *HGM04593*, *Alistipes* sp015059845, one novel species of *RUG12999*, one novel species of *Faecousia*, and one novel species of *Cryptobacteroides*. These microbial taxa were all positively correlated with greater carbohydrate. This means that the gut microbiota helps bharals to adapt to inter-region dietary differences and that higher abundance of the above microbial taxa helps bharals to adequately derive energy from their food on the diet with low crude fiber content. Inter-region differences in the metabolite composition of the microbiota indicated higher abundance of some oligosaccharides and monosaccharides in the gut microbial metabolites of bharals in the QL. This difference supports the results of inter-region differences in gut microbiota composition and function, further suggesting that gut microbiota plasticity is associated with regional dietary differences.

### Insights from wildlife conservation

The results of the bharals’ diet showed that the plant taxa that bharals mainly feed on may not be the most dominant taxa in their distribution range. This result may be the result of the bharals’ diet being mainly influenced by the composition of plants within their preferred habitat rather than the composition of plants in the whole region. The similar situation may also occur in the diet of other wild ungulates in the QTP. In future assessments of food resources and quality of ungulates in the QTP, the focus should also be on the main habitat of ungulates, rather than only from the perspective of the region as a whole.

This study was conducted during the season when bharals have access to the most abundant and nutritionally valuable food resources in the study regions. The results in this context indicated that the content of crude fiber in the diet of bharal in the QL may be lower than that in the diets of bharals in the KM and the SJY. Furthermore, bharals in the QL may need to rely on the great carbohydrate metabolism capability of their gut microbiota to meet their energy requirements under conditions of low crude fiber intake. This indicated that bharals in the QL might face challenges in food resources compared to the other two regions. Local bharal conservation should focus on the quality of bharal food resources. And in this process, it is important to focus on the quality of food within the primary range of bharal activity, rather than conducting research solely from a regional perspective. Because the edible food resources available to bharals may be concentrated within their primary range of activity, as opposed to the plants across the entire region.

Combining the results of the diet of bharals, the metabolic function of the gut bharals’ microbiota, and the metabolite composition of the gut bharals’ microbiota revealed that the carbohydrate metabolism capacity of the gut microbiota was great as the crude fiber content of the food was low. Previous studies of other ungulates in the QTP also found similar phenomena regarding the great nutrients utilization capacity of the gut microbiota in conditions of low food nutrient content. For example, the gut microbial carbohydrate metabolism capacity of Tibetan wild asses was higher in winter compared to the summer when the nutrition of the food was higher (80). In future research on ungulates in the QTP, attention should be paid to the relationship between food nutrition and nutrient utilization capacity of the hosts’ gut microbiota. And the relationship between nutrient content and the function capacity should be explored through quantitative research, rather than leaving the exploration of this content to analysis through qualitative research or extrapolation through other quantitative findings. This will facilitate further understanding of the role of gut microbiota in the adaptation of ungulates to different diets in different regions in the QTP.

## Data Availability

Raw data used in the study were uploaded to a public repository, GSA: Bioproject PRJCA027325.

## SUPPLEMENTAL MATERIAL

Fig. S1 msystems.00422-25-s0001.docx Differences in the abundance of carbohydrase genes in the bharals' microbiota among regions.

Tables S1 to S4 msystems.00422-25-s0002.xlsx Differences of microbiota among regions.

ASM does not own the copyrights to Supplemental Material that may be linked to, or accessed through, an article. The authors have granted ASM a non-exclusive, world-wide license to publish the Supplemental Material files. Please contact the corresponding author directly for reuse.
