# Supplementary material for: The plasticity of gut microbiota contributes to inter-region dietary differences adaptation of ungulates in the Qinghai-Tibet Plateau
Source: mSystems. 2025 Jul 18;10(8):e00422-25. doi: 10.1128/msystems.00422-25 (PMC12363168; doi:10.1128/msystems.00422-25)
Supplement: Fig. S1 — Differences in the abundance of carbohydrase genes in the bharals' microbiota among regions. [file msystems.00422-25-s0001.docx]

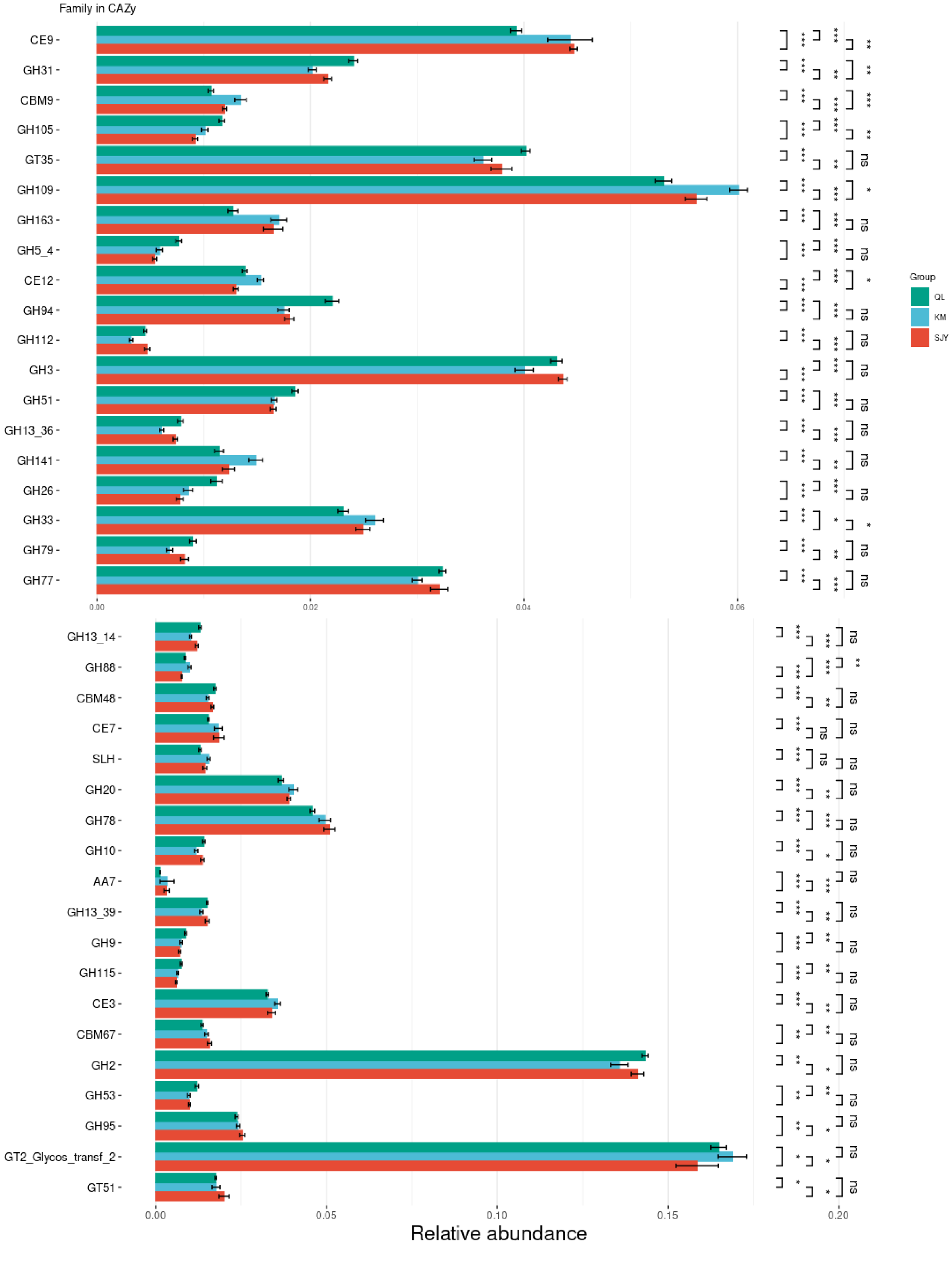


Figure S1 Differences in the abundance of carbohydrase genes in the bharals’ microbiota among regions. “ns”: *p* > 0.05, “*”: *p* < 0.05, “**”: *p* < 0.01, “***”: *p* < 0.001.
